# Long-Term Bezafibrate Treatment Improves Skin and Spleen Phenotypes of the mtDNA Mutator Mouse

**DOI:** 10.1371/journal.pone.0044335

**Published:** 2012-09-04

**Authors:** Lloye M. Dillon, Aline Hida, Sofia Garcia, Tomas A. Prolla, Carlos T. Moraes

**Affiliations:** 1 Department of Cell Biology and Anatomy, University of Miami Miller School of Medicine, Miami, Florida, United States of America; 2 Department of Neurology, University of Miami Miller School of Medicine, Miami, Florida, United States of America; 3 Department of Genetics, University of Wisconsin, Madison, Wisconsin, United States of America; University of Texas Health Science Center at San Antonio, United States of America

## Abstract

Pharmacological agents, such as bezafibrate, that activate peroxisome proliferator-activated receptors (PPARs) and PPAR γ coactivator-1α (PGC-1α) pathways have been shown to improve mitochondrial function and energy metabolism. The mitochondrial DNA (mtDNA) mutator mouse is a mouse model of aging that harbors a proofreading-deficient mtDNA polymerase γ. These mice develop many features of premature aging including hair loss, anemia, osteoporosis, sarcopenia and decreased lifespan. They also have increased mtDNA mutations and marked mitochondrial dysfunction. We found that mutator mice treated with bezafibrate for 8-months had delayed hair loss and improved skin and spleen aging-like phenotypes. Although we observed an increase in markers of fatty acid oxidation in these tissues, we did not detect a generalized increase in mitochondrial markers. On the other hand, there were no improvements in muscle function or lifespan of the mutator mouse, which we attributed to the rodent-specific hepatomegaly associated with fibrate treatment. These results showed that despite its secondary effects in rodent’s liver, bezafibrate was able to improve some of the aging phenotypes in the mutator mouse. Because the associated hepatomegaly is not observed in primates, long-term bezafibrate treatment in humans could have beneficial effects on tissues undergoing chronic bioenergetic-related degeneration.

## Introduction

Many recent studies have linked mitochondrial dysfunction with aging and aging-associated diseases [1]. The mitochondrial DNA (mtDNA) mutator mouse is a mouse model of premature aging that supports this connection. This mouse has a mutant mtDNA polymerase γ (POLG) that lacks its proofreading ability [2], [3]. Mutator mice (designated Mut mice), were shown to develop many features of premature aging such as early hair loss, anemia, osteoporosis, sarcopenia, cardiomyopathy and decreased lifespan [2], [3]. These aging-like phenotypes were associated with increased mtDNA mutation and mitochondrial dysfunction [2], [3]. We recently showed that increasing mitochondrial biogenesis and function in muscle, by transgenic overexpression of peroxisome proliferator-activated receptor (PPAR) γ coactivator-1α (PGC-1α), improved skeletal muscle and heart phenotypes of Mut mice [4].

Bezafibrate is a pharmacological agent, used in clinical practice to treat hyperlipidemia, which activates the PGC-1α/PPAR pathway [5]. It functions as a pan-agonist of the PPAR (α, δ and γ) nuclear receptor family [5]. The PPARs regulate a variety of metabolic pathways, primarily lipid metabolism; however they are also involved in regulating cell differentiation and proliferation and immune/inflammatory responses of the cell [6], [7]. Recent studies using bezafibrate to activate the PGC-1α/PPAR pathway in mouse models with mitochondrial dysfunction have yielded varied results (Table 1). Bezafibrate increased mitochondrial content and function and increased the lifespan of mice with a muscle-specific defect in cytochrome c oxidase (COX) [8]. Bezafibrate was also shown to increase PGC-1α expression and mitochondrial content/function in several tissues of a mouse model of Huntington’s disease associated with reduced PGC-1α levels [9]. In mice expressing a mutant Twinkle-helicase (deletor mice), bezafibrate reduced the number of COX negative muscle fibers, however, it did not increase mitochondrial content and function [10]. Instead, bezafibrate induced markers of fatty acid oxidation and hepatomegaly in the deletor mice [10]. In *Surf1* knock-out (KO) mice, bezafibrate also induced markers of fatty acid oxidation and hepatomegaly while increasing the expression of PPARs [11]. However, it did not induce mitochondrial content or improved the myopathy in this mouse model [11].

**Table 1:** The effect of bezafibrate treatment in mouse models of disease.

Mouse model	Bezafibrate dose	Treatment time	Results	Reference
**MLC1F-Cox10^−/−^**	Standard mouse dietwith 0.5% bezafibrate	∼2 or 5 months	- ↑ mitochondrial protein and mtDNA- ↑ COX, SDH and CS activity- ↑ % ATP in tissue- ↑ PGC-1 α and β- ↑ PPAR α, β/δ and γ- Improved mouse phenotype and survival	[8] (Wenz *et al* 2008)
***Surf1^−/−^***	Standard mouse dietwith 0.5% bezafibrate	1 month	- ↑ PPAR α and β/δ- ↑ FAO related genes (CD36, ACOX and SCAD)- No change in PGC-1α, mtDNA levels,CS or MRC activities- Weight loss- Hepatomegaly	[11] (Viscomi *et al* 2011)
**Deletor**	D12450B (rodent dietwith 10% fat) with0.5% bezafibrate	5.5 months	- No change in PGC-1α and PPARs- ↓ mtDNA copy number- Muscle : ↓ COX negative fibers; ↓ mtDNA deletion load; ↓ OXPHOS genes/proteins- Liver: hepatomegaly, ↑ FAO related genes, ↑ UCP2, ↑ FGF21- ↓ adipocyte area- Weight loss	[10] (Yatsuga *et al* 2011)
**R6/2**	Standard mouse dietwith 0.5% bezafibrate	∼2 months	- ↑ PGC-1α, PPARs and OXPHOS related genes in brain,muscle, BAT- Brain: ↑ mitochondrial density; ↓ neurodegeneration; ↓ astrogliosis; ↓ oxidative stress- Muscle: ↑ type I fibers and ↓ type IIb; restoresmitochondria structure and organization- ↓ vacuolization of BAT- Improved mouse phenotype and survival	[9] (Johri *et al* 2012)

In an attempt to compensate for mitochondrial dysfunction in the Mut mouse, we placed them on a bezafibrate diet (BD) for 8 months. Here we show that bezafibrate did not increase several tested markers of mitochondrial content/function; however, it delayed hair loss and drastically improved the skin and spleen phenotype of Mut mice. These results suggest that activation of PPARs can have beneficial effects in certain tissues undergoing mitochondrial stress.

## Results

### Systemic Effects of Bezafibrate in the Mut Mouse

To improve mitochondrial function in all tissues, 2 month-old male Mut mice were placed on a standard mouse diet containing 0.5% bezafibrate (BD). This mouse group is heretofore referred to as MutBD. To ensure that the changes observed in MutBD mice were due to bezafibrate, we also placed 2 month-old WT mice on BD (heretofore referred to as WTBD). Mice on the BD were compared to same aged male Mut and WT mice that were fed a standard mouse diet (standard diet, SD). These mice will be referred to as MutSD and WTSD respectively. The animals were fed their respective diets for 8 months and then analyzed at 10 month-old to uncover the effects of long-term bezafibrate administration on physical phenotype, organ structure/function and mitochondrial function in a model of mitochondrial aging.

**Figure 1:**
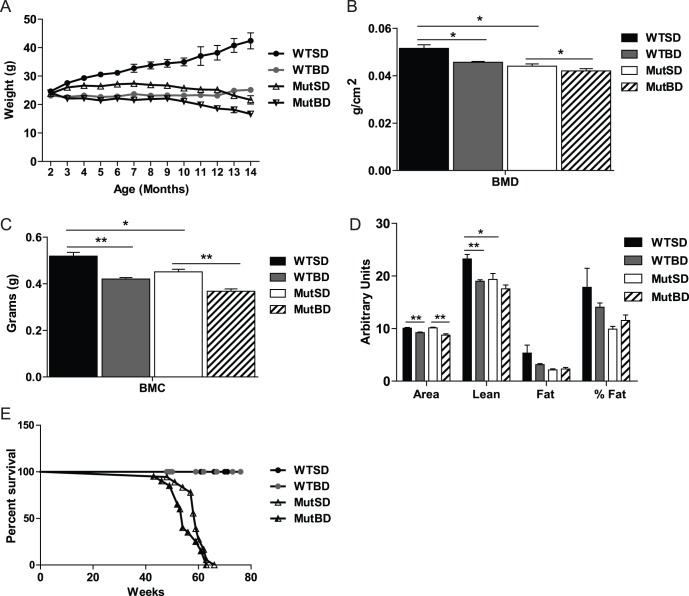
Systemic effects of bezafibrate on Mut and WT mice. (A) Body weight of mice from 2 to 14 month-old (n = 11–26/group for 2 to 11 months; 2–6/group for 12 to 14 months). The difference in body weight between WTSD and MutSD mice is statistically significant from 3 to 14 months, between WTSD and WTBD mice is significant at every time point and the difference between MutSD and MutBD is significant from 3 to 14 months. Measurement of total (B) bone mineral density (BMD), (C) bone mineral content (BMC) (D), body area (cm^2^), lean mass (g), total body fat (g) and percent (%) fat of 10 month-old mice (n = 5–7/group). (E) Percent survival of mice (n = 11–15/group). *, *P*<0.05; **, *P*<0.01, Student’s *t*-test. Error bars represent the SEM.

The Mut mouse was previously shown to have decreased body weight compared with WT mice [2], [3]. Therefore, we monitored body weight changes in both mice on BD and SD monthly beginning at 2 months of age. Similar to previous reports, we found that beginning at 3 month-older MutSD mice had a lower body weight compared with WT mice (Fig. 1A). We observed that mice in all groups had a similar body weight at 2 month-old but only WTSD mice experienced a steady increase in body weight between 4 and 11 month-old (Fig. 1A). We also found that MutBD and WTBD mice weighed less than MutSD and WTSD mice respectively beginning at 3 months of age (Fig. 1A). This difference in body weight was not due to a decrease in the food intake of MutBD mice (not shown). These results indicate that bezafibrate reduced the body weight of both Mut and WT mice.

In addition, we monitored changes in bone mineral density and content (BMD and BMC respectively), body area and body fat and lean mass of Mut and WT mice on BD and SD by DEXA scanning. Mut were previously shown to have decreased BMD, BMC and body fat at 10 month-old [2], [3]. We found that 10 month-old MutBD and WTBD mice had decreased body BMD, BMC and area compared with MutSD and WTSD mice (Fig. 1B-1D). The less dense bones may be related to lower locomotor activity or muscle function. Surprisingly, bezafibrate had no effect on fat levels in these mice (Fig. 1D).

Because the Mut has decreased lifespan compared to WT mice [2], [3], we compared the lifespan of bezafibrate treated and SD mice. Our survival studies showed that bezafibrate did not increase the percent survival of MutSD mice (Fig. 1E).

### Bezafibrate Delayed Hair Loss and Restored the Skin Structure of Mut Mice

**Figure 2:**
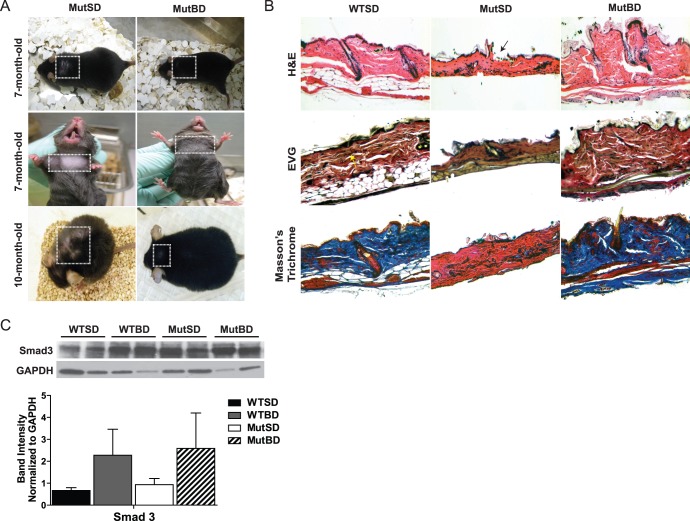
Bezafibrate delayed hair loss and restored the skin structure of Mut mice. (A) Pictures of mice at 7 month-old and 10 month-old showing their coat phenotype (n = 6/group). Squares highlight area of coat being described. (B) Dorsal skin sections from 10 month-old mice showing hematoxylin and eosin (H&E) staining to depict structural changes (black arrow indicates break in the epidermis layer of MutSD mice), Verhoeff’s Van Geison (EVG) staining for elastic fibers shown in black/dark brown (yellow arrow) and Masson’s trichrome staining showing collagen in blue (n = 2/group). (C) Western blot showing total Smad3 and glyceralgehyde 3-phosphate (GAPDH) protein levels in total skin homogenate from 10 month-old mice and quantification of total Smad3 band intensity normalized to GAPDH (n = 4/group). Error bars represent the SEM.

As part of their premature aging phenotype, Mut mice develop grey hairs and start showing signs of hair loss at about 6 to 7 months of age [2], [3]. Therefore, we compared the coat phenotype of MutBD with MutSD mice. We observed that at ∼ 7 months of age, MutSD mice started showing signs of hair loss on their back and under their neck while age matched MutBD mice that had been on the BD for 5 months did not (Fig. 2A). The hair loss in MutSD mice advanced with age, whereas MutBD mice only began showing signs of hair loss on their back at 10 month-old (Fig. 2A). In addition, we observed that 10-month-old MutBD mice had fewer gray hairs than age matched MutSD mice (not shown). These findings indicate that bezafibrate is able to delay loss and graying of the hair, two phenotypes of the Mut mice.

To further characterize the effects of bezafibrate on hair loss and graying, dorsal and ventral skin were collected from 10 month-old WTSD, MutBD and MutSD mice. Paraffin embedded skin sections were stained with hematoxylin and eosin (H&E) to determine structural changes. We observed that dorsal skin sections from MutSD mice were thinner and lacked the distinct layers that were present in WTSD mice, while MutBD mice had a skin structure more similar to WTSD mice (Fig. 2B, H&E). Specifically, the dorsal skin sections from MutSD mice were primarily composed of the dermis/connective tissue and had a non-continuous epidermis layer (Fig. 2B, H&E). In addition, the skin sections from MutSD mice had smaller hair follicles compared with WTSD and MutBD mice (not shown). No distinct structural differences were observed in ventral skin sections from these latter mice, which suggest that dorsal skin may be more susceptible to mitochondrial DNA damage. (not shown).

Because collagen and elastin fibers are the primary functional components of the dermis/connective tissue layer of the skin [12], we wanted to determine if bezafibrate had an effect on the expression of these components. To detect elastin fibers and collagen, dorsal skin sections were stained with the Verhoeff’s Van Geison (EVG) and Masson’s trichrome stain respectively. We found that elastin fibers (black/dark brown stain) were present in the connective tissue of both MutSD and MutBD mice at levels similar to WTSD mice (Fig. 2B, EVG). However, compared with WTSD mice, MutSD mice had essentially no collagen (blue stain) in the connective tissue of the skin (Fig. 2B, Masson’s trichrome). Surprisingly, the collagen levels in the skin were restored in MutBD mice (Fig. 2B, Masson’s trichrome).

The transforming growth factor beta 1 (TGF-β1)/Smad3 signaling pathway was shown to regulate a number of collagen gene promoters in human dermal fibroblasts [13]. Also, it was shown that activated PPARδ can regulate collagen levels in skin via the TGF-β1/Smad3 pathway [14]. Since bezafibrate is a PPARδ agonist, we wanted to determine whether this pathway was activated in the skin of 10 month-old MutBD mice. We performed western blot for Smad3 in total homogenate from the skin of 10 month-old mice. Our results showed an accumulation of total Smad3 protein in the skin of WTBD and MutBD mice compared with WTSD and MutSD mice (Fig. 2C). The accumulation of total Smad3 protein was previously shown to be associated with increased Smad2/3 signaling in human dermal fibroblasts [15]. Therefore, our results suggest that bezafibrate activates the TGF-β1/Smad3 pathway thereby causing an increase in collagen in the skin of MutBD mice.

### Bezafibrate Decreased Spleen Size and Restored the Spleen Structure of Mut Mice

**Figure 3:**
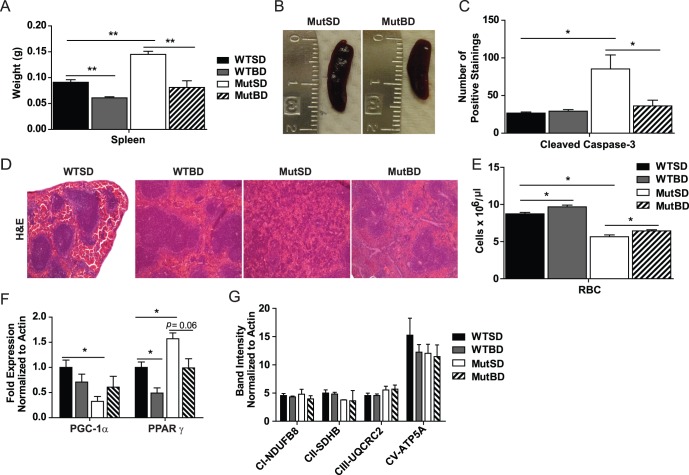
Bezafibrate improved spleen size and structure of Mut mice. (A) Spleen weight of 10 month-old mice and (B) picture of spleen from Mut mice (n = 4–6/group). (C) Quantification of cleaved caspase-3 immunostaining in paraffin sections from the spleen of 10 month-old mice (n = 3–4/group). *, *P*<0.05, one-way analysis of variance followed by Bonferroni’s multiple comparison test. (D) H&E staining of the spleen of 10 month-old mice showing the organization of white pulp (purple) and red pulp (pink) (n = 3/group). (E) Results from complete blood cell count in 10 month-old mice showing RBC (red blood cells, x10^6^µl ) (n = 5–6/group) (F) *PGC-1α* and *PPARγ* mRNA levels in the spleen of 10 month-old mice normalized to actin. (G) Quantification of western blot showing mitochondrial protein levels in total homogenate from the spleen of 10 month-old mice normalized to actin. NADH dehydrogenase (ubiquinone) 1β subcomplex subunit 8 (NDUFB8; subunit of complex I), succinate dehydrogenase subunit B (SDHB; subunits of complex II), ubiquinol-cytochrome *c* reductase core protein 2 (UQCRC2; subunit of complex III), and ATP synthase subunit 5α (ATP5A; subunit of complex V). *, *P*<0.05; **, *P*<0.01, Student’s *t*-test. Error bars represent the SEM.

Mut mice were previously shown to have enlargement of some organs, one of which was the spleen [2]. We found that bezafibrate reduced the spleen weight and size of 10 month-old MutBD and WTBD mice (Fig. 3A and 3B). The spleen size of MutBD mice was restored to that of WTSD mice (Fig. 3A). To further investigate this effect of bezafibrate on the spleen of Mut and WT mice, we performed H&E staining of paraffin embedded spleen sections. We found that bezafibrate improved the spleen structure of MutBD mice (Fig. 3D). Our results showed that MutSD mice had an aberrant spleen structure characterized by marked atrophy and decreased white pulp (purple areas in Fig. 3D) and an increase in red pulp of the spleen (Fig. 3D). As the white pulp of the spleen synthesizes antibodies, the atrophy of this area in MutSD mice can impair their immune response [16]. Interestingly, the spleen of MutBD mice had a white and red pulp distribution and organization that were similar to WTSD mice (Fig. 3D). These findings indicate that bezafibrate protected the spleen in the Mut mouse.

Because MutSD mice had pronounced atrophy in the spleen, we performed immunohistochemistry for cleaved caspase-3 in paraffin embedded spleen sections from 10-month-old mice. After quantifying the number of positive cells, we found that compared with WT mice, MutSD mice had increased cleaved caspase-3 (Fig. 3C). However, MutBD mice had reduced number of cleaved caspase-3 positive cells. (Fig. 3C), indicating that bezafibrate could reduce apoptosis in the spleen of Mut mice. This attenuation of apoptosis could explain the decreased atrophy and restoration of white pulp observed in the spleen of MutBD mice (Fig. 3D).

It was reported that Mut mice develop anemia, decreased red blood cells (RBC), which is a clinical feature in aging humans [2], [3]. The spleen is known to recycle iron from damaged RBCs and participate in extra-medullary hematopoiesis especially during anemia [16]. Because our results showed that bezafibrate improved the spleen structure of Mut mice, we wanted to determine if bezafibrate had an effect on RBC count. We performed a complete blood cell panel (CBC) in 10 month-old mice and found that while bezafibrate had no effect on white blood cell (WBC) (not shown), MutBD and WTBD mice had a moderate but significant increase in RBC count compared with MutSD and WTSD mice (Fig. 3E). However, the RBC count in MutBD mice was not restored to that of WTSD mice. These results suggest that bezafibrate increased RBC in both WT and Mut mice and we speculate that this improvement may be associated with other beneficial effects of bezafibrate in the spleen.

To further investigate the effects of bezafibrate in the spleen, we performed quantitative reverse transcriptase – polymerase chain reaction (qRT-PCR) to determine the expression level of *PPARγ* and *PGC-1α*. Both genes are known targets of bezafibrate, and PPARγ has been shown to be involved in regulating anti-inflammatory responses [17], which can influence spleen size. We found that *PPARγ* mRNA levels were elevated in the spleen of MutSD compared with WTSD mice and its levels were restored in MutBD mice (Fig. 3F). *PPARγ* levels were also reduced in WTBD mice indicating that this effect was due to bezafibrate (Fig. 3F). These findings suggest that bezafibrate may reduce inflammation and thus also the inflammatory response regulated by PPARγ in the spleen. We also found that PGC-1α levels were reduced in the spleen of MutSD mice compared with WTSD and there was a trend towards an increase in PGC-1α levels in MutBD mice (Fig. 3F). To determine if this mild increase in PGC-1α levels caused an increase in mitochondrial content in the spleen of MutBD mice, we performed western blot to assess mitochondrial protein levels in total spleen homogenate. We did not observe an increase in mitochondrial protein levels in bezafibrate treated mice. (Fig. 3G). However, it is possible that some cell types in the spleen may have increased mitochondrial protein levels in response to bezafibrate that was undetectable in total spleen homogenate. These results indicate that beneficial effects of the bezafibrate in the spleen are not due to a robust increase in mitochondrial content but perhaps due to reduced apoptosis and anti-inflammatory responses regulated by PPARγ.

### Bezafibrate Induced Fatty Acid Oxidation but did not Increase Global Mitochondrial Content in the Skeletal Muscle of Mut Mice

**Figure 4:**
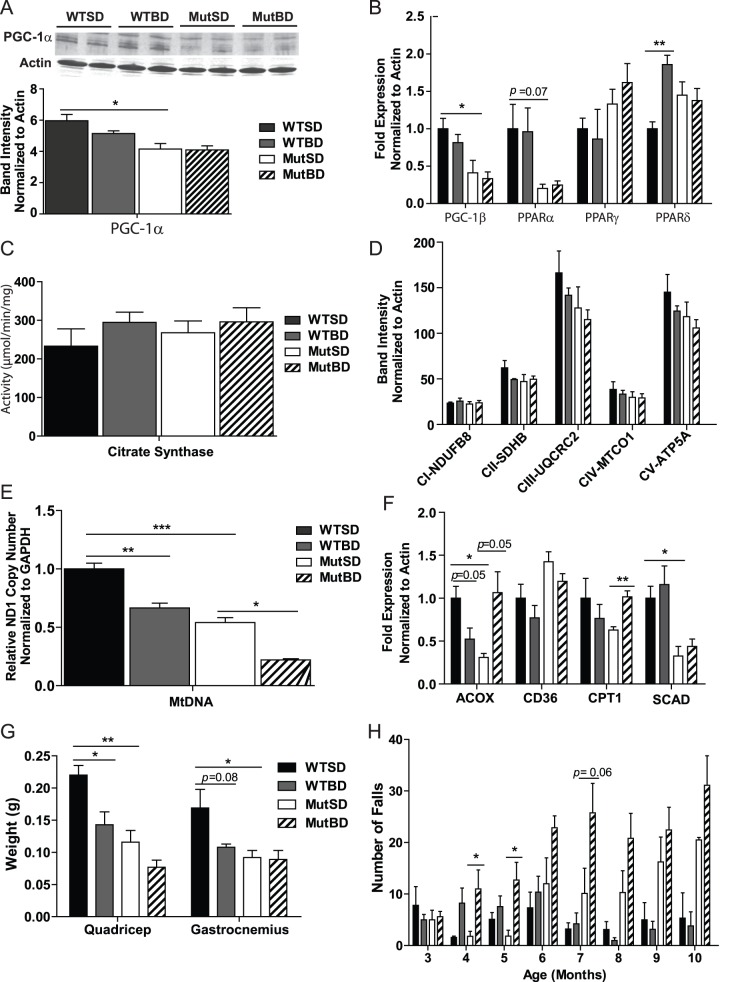
The effect of bezafibrate on the skeletal muscle of Mut and WT mice. (A) Western blot of PGC-1α and loading control actin in total quadricep homogenate from 10 month-old mice and quantification of PGC-1α band intensity normalized to actin (n = 4/group). (B) Gene expression of *PGC-1β* and *PPARs* in the quadricep of 10 month-old mice normalized to actin (n = 4/group). (C) Citrate synthase activity in the total quadricep homogenate from 10 month-old mice (n = 4/group). (D) Quantification of western blot of mitochondrial proteins in the total homogenate from the quadricep of 10 month-old mice (n = 4/group). NADH dehydrogenase (ubiquinone) 1β subcomplex subunit 8 (NDUFB8; subunit of complex I), succinate dehydrogenase subunit B (SDHB; subunits of complex II), ubiquinol-cytochrome *c* reductase core protein 2 (UQCRC2; subunit of complex III), mitochondrial cytochrome c oxidase subunit 1 (MTCO1; subunit of complex IV) and ATP synthase subunit 5α (ATP5A; subunit of complex V) (E) Quantification of mtDNA levels in the quadriceps of 10 month-old mice based on ND1 (subunit of complex I) levels normalized to glyceraldehyde 3-phosphate (GAPDH) (n = 4/group). (F) Gene expression of markers of fatty acid oxidation, acyl-coenzymeA oxidase 1 (ACOX), cluster of differentiation 36 (CD36), carnitine palmitoyl transferase (CPT1) and short-chain-acyl-coenzymeA dehydrogenase (SCAD) in the quadriceps of 10 month-old mice normalized to actin (n = 4/group) (G) Skeletal muscle weight of 10 month-old mice (n = 4–6/group). (H) The number of falls of mice when put to run on a treadmill for 3 minutes at 9 meters/minute (n = 5–10/group). *, *P*<0.05; **, *P*<0.01, ***P<0.001, Student’s *t*-test. Error bars represent the SEM.

As part of their premature aging phenotype, Mut mice develop sarcopenia and mitochondrial dysfunction in the skeletal muscle [18]. To determine if bezafibrate was able to induce mitochondrial content/function in the skeletal muscle of Mut mice, we performed western blot to detect the levels of PGC-1α and mitochondrial proteins in total quadricep homogenate from 10 month-old mice. We found that MutSD mice had reduced PGC-1α protein levels compared with WTSD mice and the levels were not restored in MutBD mice (Fig. 4A). Similarly, MutSD mice had reduced *PGC-1β* mRNA levels compared with WTSD mice and these levels were also not increased in MutBD mice (Fig. 4B). Also, bezafibrate did not increase mitochondrial protein levels or citrate synthase activity (CS) in the quadriceps of MutBD and WTBD mice (Fig. 4C and 4D respectively). We also did not detect an increase in cytochrome c oxidase (COX) activity in total quadriceps homogenate of MutBD when compared to MutSD mice (not shown). To further characterize the effects of bezafibrate on mitochondria in the skeletal muscle, we quantified mtDNA levels in the quadricep by qPCR. Paradoxically, we found that MutBD and WTBD mice had decreased mtDNA levels compared with MutSD and WTBD (Fig. 4E). These results indicate this bezafibrate regimen did not induce mitochondrial content in the skeletal muscle of Mut and WT mice.

To determine the effect of bezafibrate on the expression of PPARs in the skeletal muscle, we performed qRT-PCR and found that bezafibrate had no effect on the mRNA levels of PPARs in the skeletal muscle of Mut mice (Fig. 4B). However, we found that the expression of some fatty acid oxidation-related genes, known to be regulated by the PPARs, was increased in the skeletal muscle (Fig. 4F). The mRNA levels of acyl-coenzymeA oxidase 1 (ACOX), an enzyme that oxidizes long chain and branched fatty acids, and carnitine palmitoyl transferase (CPT1), enzyme involved in fatty acid transport into mitochondria, were elevated in the quadriceps of MutBD mice (Fig. 4F). Interestingly, these increases were not found in WTBD mice (Fig. 4F). In addition, there was no change in the mRNA levels of cluster of differentiation 36 (CD36), an integral membrane protein that internalizes lipoproteins and fatty acids, and short-chain-acyl-coenzymeA dehydrogenase (SCAD), an enzyme that metabolizes short-chain fatty acids (Fig. 4F). These results suggest that bezafibrate increased mitochondrial fatty acid oxidation in the skeletal muscle of MutBD mice.

### Bezafibrate did not Improve the Skeletal Muscle Weight or Function of Mut Mice

Because Mut mice were shown to have loss of skeletal muscle mass [18], we also monitored the effect of bezafibrate on skeletal muscle weight. We found that while WTBD mice had reduced quadriceps and gastrocnemius weight compared with WTSD mice, bezafibrate had no effect on the weight of these muscles in MutBD mice (Fig. 4G). Nevertheless, we went on to compare the motor function/skeletal muscle function of MutBD and WTBD mice to that MutSD and WTSD mice by assessing their performance on a treadmill test. We began testing mice at 3 months of age and monitored their performance monthly. Our results showed that between 4–6 months of age both MutBD and WTBD mice fell more than MutSD and WTSD mice (Fig. 4H). This trend continued between 7–10 months of age for MutBD (Fig. 4H). These results indicate that bezafibrate did not prevent the development of sarcopenia or improved the skeletal muscle function of Mut mice.

### Bezafibrate Induced Hepatomegaly and Fatty Acid Oxidation in the Liver of Mut and WT Mice

**Figure 5:**
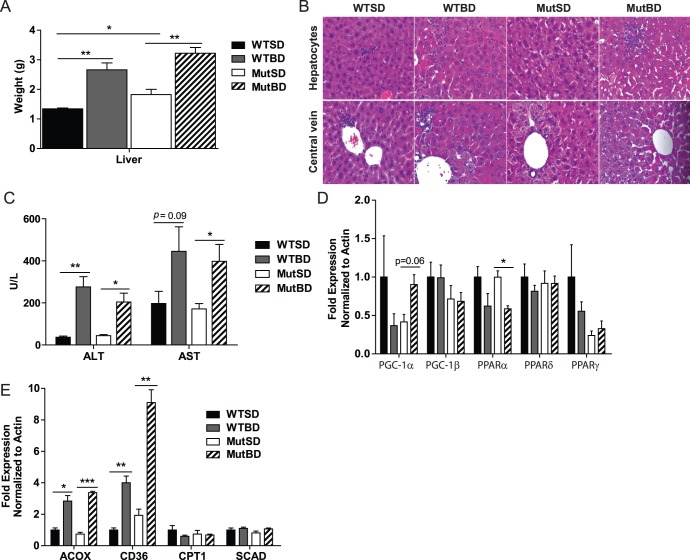
Bezafibrate induces hepatomegaly and fatty acid oxidation in Mut and WT mice. (A) Liver weight of 10 month-old mice (n = 4–6/group). (B) H&E staining of the liver of 10 month-old mice showing hepatocytes and central vein (n = 4/group). (C) The level of liver enzymes alanine aminotransferase (ALT) and aspartate aminotransferase (AST) in the blood of 10 month-old mice (n = 6/group). (D) Gene expression of *PGC-1* coactivators and *PPARs* in the liver of 10 month-old mice normalized to actin (n = 4/group). (E) The mRNA level of markers of fatty acid oxidation in the liver of 10 month-old mice normalized to actin (n = 4/group). Acyl-coenzymeA oxidase 1 (*ACOX*), cluster of differentiation 36 (*CD36*), carnitine palmitoyl transferase (*CPT1*) and short-chain-acyl-coenzymeA dehydrogenase (*SCAD*). *, *P*<0.05; **, *P*<0.01, ***P<0.001, Student’s *t*-test. Error bars represent the SEM.

Bezafibrate was previously shown to have hepatoproliferative effects on rodent liver [10], [19]. Therefore, we monitored the liver phenotype of our mice. We found that bezafibrate doubled the liver weight of 10 month-old MutBD and WTBD mice compared to MutSD and WTSD mice (Fig. 5A). To determine whether there was a liver pathology, the liver of MutBD and WTBD and standard controls were subjected to histological analysis. H&E staining of the liver showed that MutBD and WTBD mice seemed to have enlarged hepatocytes around central veins of the hepatic lobules, consistent with the observed hepatomegaly (Fig. 5B). Furthermore, we performed a hepatic panel and found that 10 month-old MutBD and WTBD had significantly increased levels of liver enzymes aspartate aminotransferase (AST) and alanine aminotransferase (ALT) in their blood compared to MutSD and WTSD mice, a sign of liver damage (Fig. 5C). These results indicate that bezafibrate induced hepatomegaly and liver damage in Mut and WT mice.

To determine the effect of bezafibrate on the RNA levels of PGC-1α and β, as well as PPARs in the liver of Mut and WT mice, we performed qRT-PCR. We found that bezafibrate had no effect on the mRNA levels of PPARδ, PPARγ and PGC-1β in liver of 10 month-old mice (Fig. 5D). However, bezafibrate reduced the mRNA levels of PPARα in both MutBD and WTBD mice and we found a trend towards increased mRNA for PGC-1α levels in the liver of MutBD mice (Fig. 5D). To determine if this increase in PGC-1α RNA resulted in increased mitochondrial content in the liver, we performed western blot to detect the levels of mitochondrial protein. We found that bezafibrate did not increase the levels of mitochondrial proteins in the liver of MutBD and WTBD mice (not shown). Finally, it was recently shown that bezafibrate induces fatty acid oxidation in the liver of mice [10]. We confirmed this by performing qRT-PCR. Our results shown that bezafibrate increased the mRNA levels of *ACOX* and *CD36* in the liver of both MutBD and WTBD mice (Fig. 5E). However, there was no change in the gene expression of *CPT1* and *SCAD* (Fig. 5E). These results indicate that bezafibrate induced fatty acid oxidation in the liver of Mut and WT mice. However, further experiments are needed to confirm this finding.

## Discussion

We hypothesized that bezafibrate would induce increased mitochondrial content in the Mut mouse and the latter would ameliorate some of the premature aging phenotypes observed in these mice. Our results showed a more complex picture, where beneficial effects were observed for some tissues, but not others. Moreover, the beneficial effects were not clearly linked to increased mitochondrial content.

In the skin, the most striking finding was the restoration of collagen protein in the dermis of MutBD mice. Since decreased collagen synthesis is a feature of skin aging [12], bezafibrate seemed to have protected the skin in Mut mice from premature aging-like phenotypes. Furthermore, because collagen is an important structural component of the dermis skin layer where the hair follicles are found, restoration of collagen levels may have contributed to the preservation of hair follicles and the delay in hair loss we observed in MutBD mice.

Although we were surprised by the beneficial effects of bezafibrate in the skin; several studies have highlighted the importance of PPARs in skin biology [7]. PPARs have been shown to play a role in skin permeability, epidermal cell growth/differentiation, skin inflammatory response and wound healing [7]. In fact, activation of PPARδ was shown to be able to influence collagen expression and promote wound healing in the skin via TGF-β1/Smad3 signaling [14]. We showed that bezafibrate treated mice had an accumulation of total Smad3 protein in the skin, an indicator of increased Smad2/3 signaling [15], suggesting that this pathway may have been activated by bezafibrate to promoting an increase in collagen levels.

We also showed that mice on the BD had reduced spleen weight and restoration of the red and white pulp organization in the spleen. The increased cleaved caspase-3 positive staining detected in the spleen of MutSD mice was not surprising since Mut mice were shown to have increased apoptosis in many tissues [3]. However, we found that MutBD mice had less cleaved caspase-3 positive staining; suggesting that bezafibrate was reducing apoptosis in the spleen. We also found that bezafibrate treated mice had increased red blood cell count which may have resulted from the restoration of spleen structure. In addition, *PPARγ* mRNA levels were lower in the spleen of MutBD mice. Studies have shown that PPARγ controls inflammation by inhibiting the expression of inflammatory cytokines [6], [7]. Therefore, we speculate that *PPARγ* levels were increased in MutSD mice to promote an anti-inflammatory response and this response was no longer needed in MutBD mice and so *PPARγ* levels were reduced.

Similar to previous findings in deletor [10] and *Surf1* KO mice [11], bezafibrate did not induce increased mitochondrial content/function in skeletal muscle of Mut mice. Interestingly, we found that PGC-1α protein levels were reduced in the skeletal muscle of Mut mice and they were not restored by bezafibrate. This raises the question of whether reduction in PGC-1α contributes to the skeletal muscle phenotype of Mut mice. Nevertheless, we found that bezafibrate increased markers of fatty acid oxidation in the skeletal muscle, similar to what was shown in *Surf1* KO mice [11]. However, while bezafibrate increased the expression of PPARs in the skeletal muscle of *Surf1* KO mice [11], PPAR expression was either decreased or unaffected in all the tissues we analyzed in bezafibrate treated Mut mice. We cannot eliminate the possibility that bezafibrate increased PPAR expression in Mut mice, but this was not observed when we analyzed 10 month-old mice on BD for 8 months.

Our laboratory previously showed that bezafibrate increased *PGC-1α* expression and mitochondrial content in MLC1F-Cox10^−/−^ mice thereby delaying the onset of the myopathy [8]. However, we could not demonstrate a generalized increase in mitochondrial content in the Mut mice, although the expression of some genes coding for mitochondrial proteins associated with β-oxidation were increased. We suspect that this difference in effect of bezafibrate in the muscle of Mut mice compared to MLC1F-Cox10^−/−^ mice may be due to the low levels of PGC-1α in the muscle of the Mut mouse. Another possibility is that this discrepancy may be due to the difference in bezafibrate treatment time. Long-term bezafibrate treatment in mutator mice could have exacerbated the toxic effects of bezafibrate on the liver, which in turn would reduce the potential benefits of bezafibrate on the muscle. We previously showed that transgenic expression of PGC-1α in the skeletal muscle of Mut mice increased mitochondrial content and function and improved mouse treadmill performance [4]. Taken together, these findings showed that overexpression of PGC-1α confers more benefits to the skeletal muscle of Mut mice than bezafibrate administration.

In this study, we confirmed that bezafibrate has a toxic effect on mouse liver; it induced hepatomegaly in both WT and Mut mice. This is a rodent specific effect of bezafibrate that was also observed in the deletor and *Surf1* KO mice [10], [11]. In addition, bezafibrate induced markers of fatty acid oxidation in the liver of both WT and Mut mice, a pathway known to be regulated by PPARs [6]. Interestingly, we observed that bezafibrate induced an increase in CD36, a marker of fatty acid oxidation, in the liver but not in the skeletal muscle. Furthermore, we found that bezafibrate treated WT and Mut mice had decreased body weight, likely because of the reduced BMD, BMC and body area. We also observed that bezafibrate caused a greater decrease in body weight in WT than Mut mice. This difference may be due to reduced weight of the Mut mice compared to the WT or a partial compensatory effect of the bezafibrate treatment in the Mut mice. In addition, we suspect that decreased locomotion observed in bezafibrate treated mice is related to the effects of bezafibrate on BMD and BMC. We speculate that these non-beneficial effects of bezafibrate on the overall well-being of the mice may have obscured its pharmacological beneficial effects. The early death of MutBD mice may be the result of the liver damage they sustained in combination with the phenotypes that were not ameliorated by bezafibrate. It is important to note that the concentrations of bezafibrate used here are approximately 80-fold higher than what is used to treat dyslipidemias in humans [20] highlighting the need to study the effect of bezafibrate on mitochondrial function in humans with mitochondrial defects.

Here we showed that bezafibrate improved some premature aging-like phenotypes of Mut mice, most clearly observed in skin and spleen. Recently, it was shown that mitochondrial dysfunction in somatic stem cells may contribute to the progeroid phenotypes present in Mut mice [21]. Therefore, our findings suggest that bezafibrate may help maintain the stem cell population in replicating tissues like skin and spleen in the Mut mice. Interestingly, the benefits observed did not correlate with generalized increased mitochondrial content or function, and may also result from the effects of PPARs in other related regulatory pathways involved in the control of inflammation, fatty acid metabolism and apoptosis.

## Materials and Methods

### Mouse Model

MtDNA mutator mice (Polg^D257A/D257A^) (Mut) were previously characterized [2], [3]. We placed 2 month-old male Mut and WT mice (MutBD and WTBD respectively) on a standard mouse diet containing 0.5% bezafibrate (bezafibrate diet, BD) (Bio-Serv). As a control, we placed same age male Mut and WT mice on the standard mouse diet (standard diet, SD) (MutSD and WTSD respectively). Mice were kept on their respective diets for 8 months and analyzed at 10 month-old. We choose to use only male mice to reduce the total number of animals used for our study and also to avoid experimental variability observed in female animals due to their estrous cycle.

### Animal Husbandry

Mice were housed in a virus-antigen-free facility of the University Of Miami Division Of Veterinary Resources in a 12-h light/dark cycle at 22°C and fed *ad libitum* with irradiated standard mouse diet or bezafibrate diet.

### DEXA Scan

This procedure was performed as previously described [22]. In summary, mice were anesthetized then weighed and bone mineral density and content along with fat and lean body mass was measured using a Lunar PIXImus II Densitometer (GE Medical Systems, Waukesha, WI).

### Histology

The dorsal and ventral skin of euthanized mice was shaved, and skin biopsies were collected and stored in formalin for at least 24 hours before being sent to the University of Miami Lois Pope Life Centre histology core for paraffin embedding. Paraffin skin sections were stained with hematoxylin and eosin (H&E), Verhoeff’s Van Geison (EVG) and Masson’s trichrome stain at the University of Miami Dermatology Pathology Core. The stained sections were analyzed using a light microscope. For spleen and liver analysis, deeply anesthetized mice were perfused with cold PBS and the liver and spleen were collected and stored in formalin for at least 24 hours. Fixed tissues were then paraffin embedded, sectioned, stained for H&E and analyzed for structural changes at the University of Miami Comparative Pathology Laboratory.

### Immunostaining

Paraffin embedded spleen sections were warmed then deparaffinized and rehydrated. Sections were incubated in 70% formic acid to retrieve antigen before being permeabilized with 0.2% Triton. Endogenous peroxidases were quenched then sections were blocked for 1 hour with normal goat serum (KPL). Sections were incubated overnight in 1∶250 dilution of primary antibody for cleaved caspase-3 (Cell Signaling). Secondary antibody, biotinylated goat anti-rabbit (KPL), was used then sections were treated with streptavidin peroxidases (KPL) for 30 minutes before being incubated in Diaminobenzidine (DAB). Slides were dehydrated in alcohol then mounted in xylene with aqueous mounting medium and viewed with a light microscope. The number of positive (brown) staining was counted in 4 field of view from each section and averaged.

### Western Blot

Western blot was performed as previously described [23]. Primary antibodies used were Smad3 (Cell Signaling), GAPDH (GeneTex), Total OXPHOS Rodent Cocktail (Mitosciences), PGC-1α (Santa Cruz H-300), and Actin (Sigma). Primary antibodies were used at 1∶1000 dilutions, except PGC-1α which was used at 1∶200 dilution, and incubated overnight at 4°C. Secondary antibodies used were infrared-conjugated anti-rabbit 700 (1∶3000) and anti-mouse 800 (1∶5000) (Rockland) and goat anti-rabbit HRP-linked (1∶1000) (Cell Signaling). Blots were either visualized with Odyssey Infrared Imaging System (LI-COR Biosciences) and band intensity quantified with default software supplied by LI-COR or they were visualized with X-ray film developer and band intensity quantified with ImageJ software.

### Quantitative PCR

Total RNA was isolated from snap frozen tissue using the RNeasy Fibrous Tissue Mini kit (for quadriceps) and RNeasy Mini kit (for liver and spleen) (Qiagen). cDNA was synthesized from 1µg of RNA using the iScript cDNA synthesis kit (BIO-RAD). Quantitative real-time PCR, with specific primers for PGC-1α (5′-CTGCGGGATGATGGAGACA, 5′-AGCAGCGAAAGCGTCACA), PGC-1β (5′- TGGCCCAGATACACTGACTATG,

5′- TGGGCCTCTTTCAGTAAGCT), PPARα (5′- TTCCCTGTTTGTGGCTGCTAT,

5′- CCCTCCTGCAACTTCTCAATGTAG), PPARγ (5′- CGGAAGCCCTTTGGTGACTTTA,

5′- GCGGTCTCCACTGAGAATAATGAC), PPARδ (5′- ACCGCAACAAGTGTCAGTAC, 5′- CTCCGGCATCCGTCCAAAG), ACOX (5′- ACCGCCTATGCCTTCCACTTTC, 5′- GCAAGCCATCCGACATTCTTCG), CD36 (5′- CCAAATGAAGATGAGCATAGGACAT, 5′-GTTGACCTGCAGTCGTTTTGC), CPT1 (5′- TTTCGACAGGTGGTTTGAC, 5′- TCTGCGTTTATGCCTATCTTG), SCAD (5′- CCTGGATTGTGCTGTGAA, 5′- TGTCTGCCAGCTTGAACT) and β-Actin (5′-TGACAGGATGCAGAAGGAGAT,

5′-GCGCTCAGGAGGAGCAAT) was performed using SsoAdvanced SYBR Green (BIO-RAD). MtDNA copy number was quantified as previously described [4]. ΔΔCt method was used to determine the relative abundance of each gene.

### Spectrophotometric Assays

Citrate synthase (CS) activity was determined spectrophotometrically in total homogenate from the quadriceps of mice as previously described [4]. Assay results were normalized to protein concentration obtained by the Bradford method.

### Treadmill Test

The treadmill test was performed as previously described [4]. Mice were first acclimated to the treadmill (Columbus Instruments, Columbus, OH) then they were put to run at 9 meters per minute for 3 minutes. Mice performance was determined by the number of times a mouse falls off the running belt onto the grid during the test.

### Blood Analysis

Mice were fasted overnight and the following day anesthetized and blood was collected by cardiac puncture. Blood was sent to the University of Miami Comparative Pathology Laboratory for analysis of complete blood cell count (CBC) and for a hepatic blood panel to measure markers of liver damage/inflammation.

### Ethics Statement

This study was carried out in strict accordance with the recommendations in the Guide for the Care and Use of Laboratory Animals of the National Institutes of Health. The protocol was approved by the Committee on the Ethics of Animal Experiments of the University of Miami (Permit Number: 12-094). All efforts were made to minimize suffering.
